# Combined β-sitosterol and trimetazidine mitigate potassium dichromate-induced cardiotoxicity in rats through the interplay between NF-κB/AMPK/mTOR/TLR4 and HO-1/NADPH signaling pathways

**DOI:** 10.1007/s11356-023-27021-1

**Published:** 2023-04-28

**Authors:** Ehab A. M. El-Shoura, Maha A. Salem, Yasmine H. Ahmed, Lamiaa Khalaf Ahmed, Dalia Zaafar

**Affiliations:** 1 Department of Pharmacology and Toxicology, Faculty of Pharmacy, Al-Azhar University, Assiut Branch Assiut, 71524 Egypt; 2 Department of Pharmacology and Toxicology, Faculty of Pharmacy, Modern University for Technology, and Information, Cairo, Egypt; 3 Department of Cytology and Histology, Faculty of Veterinary Medicine, Cairo University, Giza, Egypt; 4 Department of Biochemistry and Molecular Biology, Faculty of Pharmacy (Girls), Al-Azhar University, Cairo, 71524 Egypt

**Keywords:** Potassium dichromate, β-sitosterol, Trimetazidine, Cardiotoxicity, Oxidative stress, Inflammation

## Abstract

**Abstract:**

Hexavalent chromium salt, like potassium dichromate (PD), is chromium’s most precarious valence state in industrial wastes. Recently, there has been increasing interest in β-sitosterol (BSS), a bioactive phytosterol, as a dietary supplement. BSS is recommended in treating cardiovascular disorders due to its antioxidant effect. Trimetazidine (TMZ) was used traditionally for cardioprotection. Through the administration of BSS and TMZ, the cardiotoxic effects of PD were to be countered in this study, in addition to examining the precise mechanism of PD-induced cardiotoxicity. Thirty male albino rats were divided into five groups; the control group: administered normal saline daily (3 mL/kg); the PD group: administered normal saline daily (3 mL/kg); BSS group: administered BSS daily (20 mg/kg); TMZ group: administered TMZ daily (15 mg/kg); and the BSS + TMZ group: administered both BSS (20 mg/kg) and TMZ (15 mg/kg) daily. All experimental groups, except the control, received on the 19th day a single dose of PD (30 mg/kg/day, S.C.). Normal saline, BSS, and TMZ were received daily for 21 consecutive days p.o. The exposure to PD promoted different oxidative stresses, pro-inflammatory, and cardiotoxicity biomarkers. BSS or TMZ succeeded solely in reducing these deleterious effects; however, their combination notably returned measured biomarkers close to normal values. The histopathological investigations have supported the biochemical findings. The combination of BSS and TMZ protects against PD cardiotoxicity in rats by reducing oxidative stress and apoptotic and inflammatory biomarkers. It may be promising for alleviating and protecting against PD-induced cardiotoxicity in people at an early stage; however, these findings need further clinical studies to be confirmed.

**Highlights:**

• Potassium dichromate induces cardiotoxicity in rats through the upregulation of oxidative stress, proinflammatory, and apoptotic pathways biomarkers.

• β-Sitosterol possesses a possible cardioprotective effect by modulating several signaling pathways.

• Trimetazidine, the antianginal agent, has a potential cardioprotective impact on PD-intoxicated rat model.

• The combination of β-Sitosterol and trimetazidine was the best in modulating different pathways involved in PD cardiotoxicity in rats via the interplay between NF-κB/AMPK/mTOR/TLR4 and HO-1/NADPH signaling pathways.

**Graphical Abstract:**

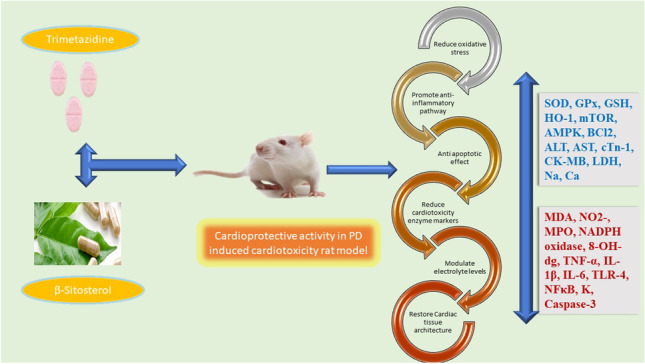

## Introduction

It is well known that exposure to chromium compounds in the environment and occupation, especially hexavalent chromium like potassium dichromate (PD), may be toxic to both humans and animals. It is a xenobiotic agent and a heavy metal linked to multiple organ toxicity. Many cellular metabolites reduce PD to produce chromium, leading to the overproduction of reactive oxygen species (Salama et al. 2022). The overproduction of free radicals is thought to be linked to its toxicity by causing oxidative damage to the tissues of the liver, brain, heart, and kidneys. Also, chromium causes lipid peroxidation, DNA damage, and the production of numerous pro-inflammatory biomarkers that contribute to the inflammatory process and ultimately cause severe nephrotoxicity (Mehany et al. 2013).

Cr(VI) is classified as a redox-active metal because it undergoes direct redox cycling, producing a large amount of ROS (Stohs and Bagchi 1995). Excessive ROS production leads to calcium (Ca^2+^) and sodium (Na^+^) overload. Excessive ROS production can also damage mitochondrial and other organelle membranes, resulting in cellular Ca^2+^ overload due to Ca^2+^ influx from calcium stores (Görlach et al. 2015). It has been reported that PD inhibits the activities of Ca^2+^/Mg^2+^-ATPase and Na^+^/K^+^-ATPase, resulting in Ca^2+^ and Na^+^ overload (Ghosh and Dey 2022).

The transcriptional factor NF-ĸB regulates the expression of various pro-inflammatory genes and plays a role in inflammasome regulation. All of NF-ĸB actions are mediated by the upregulation of cytokines and chemokines such as tumor necrosis factor-alpha (TNF-α), interleukins (IL-1β, and IL-6), which regulate the activation, differentiation, and survival of innate immune cells (Liu et al. 2017). PD significantly increases the activity of NF-ĸB in various rat tissues, which initiates inflammatory cascades by releasing a wide range of pro-inflammatory cytokines (Anandasadagopan et al. 2017).

AMP-activated protein kinase (AMPK) is a protein kinase that is AMP dependent. AMPK regulates cellular energy synthesis and catabolism. It is also an essential cell growth, reproduction, and autophagy regulator (Schmitt et al. 2022). AMPK is a crucial receptor for cellular energy. Cr(VI) can, through mitigating the AMPK/peroxisome proliferator-activated receptor-γ coactivator signaling pathway, leads to its apoptosis and mitochondrial dysfunction (Yang et al. 2020).

The mechanistic target of rapamycin is one of the PI3K-related kinase family members and a serine/threonine kinase (Wander et al. 2011). It is a key integrator of signals that control the biosynthesis of both protein and lipid, as well as a growth factor–driven cell cycle progression and a key regulator of protein synthesis in the cardiomyocyte (McMullen et al. 2004). Reduced mTOR activity has been linked to doxorubicin-induced cardiac dysfunction.

Trimetazidine is a piperazine-derived anti-angina agent. It has numerous cardioprotective effects, such as lowering vascular resistance, promoting cardiac cell metabolism and blood circulation, and regaining the capacity for generating energy (Tsioufis et al. 2015; Dézsi 2016). Previous research showed that TMZ has anti-oxidation, anti-apoptosis, and anti-inflammation properties, as well as the capacity to enhance myocardial metabolism and energy production (Zou et al. 2017). Additionally, a prior study suggested that the early administration of TMZ could reduce myocardial fibrosis and apoptosis, effectively alleviating diabetic cardiomyopathy (Zhang et al. 2016). Numerous studies demonstrated how TMZ protects against cardiovascular disease by reducing oxidative stress, apoptosis, and inflammation (Zhang et al. 2019; Chen et al. 2022). However, data on the effects of TMZ pre-treatment on PD cardiotoxicity are limited, and the underlying mechanisms remain unknown.

The phytosterol BSS, present in various plants, resembles cholesterol chemically (Kasirzadeh et al. 2021). Through its anti-inflammatory effect on human aortic endothelial cells, BSS has been demonstrated to be a potential agent for preventing atherosclerosis (Loizou et al. 2010). Similarly to this, it has been reported that BSS inhibits inflammatory biomarkers like IL-6 and TNF-α in human monocytes that have been activated by endotoxin (Loizou et al. 2010). Although sepsis and neurological impairment had been studied among several models for the positive effects of BSS, there was little information regarding the heart (Chandra et al. 2022; Kasirzadeh et al. 2021).

### Aim of the work

Here, we proposed that TMZ and BSS might be useful in reducing myocardial damage following the induction of cardiotoxicity. In the current study, we established a rat model of PD-induced cardiotoxicity. The rats received TMZ, BSS, or a combination of the two treatments before receiving a subcutaneous injection of PD. We aimed to learn more about how the compounds under investigation affected cardiac injury and the cellular and molecular mechanisms underlying it.

## Materials and methods

### Chemicals and reagents

PD and BSS were acquired from Sigma Aldrich (St. Louis, MO, USA). TMZ was purchased from GNP pharmaceutical (Giza, Egypt).

### Animals and study design

Thirty male albino rats, 10 weeks old, weighing 170–180 g, were obtained from animal house of Faculty of Medicine, Assiut University. Rats were housed in polypropylene cages (at 25 °C under a normal light/dark cycle) for 7 days prior to the start of the experimental protocol to acclimate them to the controlled laboratory conditions. Rats were fed a standard rodent diet and had unrestricted access to clean water. All experimental procedures adhered to the National Institutes of Health Guidelines for the Care and Use of Laboratory Animals. The Faculty of Medicine approved the experimental protocol at Assiut University’s Institutional Animal Ethics Committee (approval no: 17300797).

Rats were divided into the following five groups by random allocation following the 7-day acclimatization period (*n* = 6):

- Group 1: Control group: administered daily oral normal saline solution (3 mL/kg) for 21 days.
- Group 2: PD group: administered daily oral normal saline solution (3 mL/kg) for 21 days. PD (30 mg/kg/day, S.C.) was dissolved in normal saline and received a single dose of PD on day 19 of the experiment according to the methodology of Awoyomi et al. (2021).
- Group 3: BSS group: administered a daily oral dose of BBS (20 mg/kg, p.o.) for 21 consecutive days and received a single dose of PD on day 19 of the experiment (30 mg/kg/day, S.C.). BSS was received according to Gumede and colleagues’ methodology (Gumede et al. 2020).
- Group 4: TMZ group: administered a daily oral dose of TMZ (15 mg/kg, p.o.) for 21 consecutive days following Ussher and colleagues’ method (Ussher et al. 2014). PD was received as a single dose on day 19 of the experiment (30 mg/kg/day, S.C.).
- Group 5: BSS + TMZ group: administered daily oral doses of both BBS (20 mg/kg, p.o.) and TMZ (15 mg/kg, p.o.) for 21 consecutive days. PD was received as a single dose on the 19th day of the experiment (30 mg/kg/day, S.C.).

### Blood and tissue sampling

After the 21st day of the experimental study, blood samples were gathered from the inner canthus of the eye of each rat and centrifuged for 15 min at 3000 rpm to collect the separated sera, which were preserved at – 80 °C until quantifications of cardiotoxicity biomarkers and electrolyte levels were performed. Then, rats ted. Then rats were euthanized by anesthesia with a mixture of ketamine (100 mg/kg, i.p.) and xylazine (10 mg/kg, i.p.). Hearts were quickly removed, washed using cold saline, and preserved in either a − 80 °C deep freezer for western blot analysis or neutral buffered formalin (10%) for histopathology and immunohistochemistry. Cardiac tissue homogenates were prepared (20% w/v) in ice-cooled phosphate-buffered saline using homogenizer and centrifuged for 15 min at 5000 × g at 4 °C. Tissue homogenates were divided into aliquots and stored at – 80 °C until quantification of oxidative stress biomarkers and pro-inflammatory mediators. Fixed samples were dehydrated, followed by xylene, and embedded in paraffin. Sections 3-μm thick were prepared, deparaffinized, and stained with hematoxylin and eosin (H&E) (Banchroft and Steven 1983).

### Estimation of oxidative stress biomarkers in cardiac tissue

Lipid peroxidation was accurately measured chemically using a colorimetric method. Malondialdehyde (MDA) was measured in the heart tissue as a lipid peroxidation marker. In a nutshell, the malondialdehyde content of cardiac cells forms thiobarbituric acid reactive product tissue after reacting with thiobarbituric acid. This product can be measured by measuring the solution’s absorbance spectrophotometrically at wavelength of 520–535 nm. Following the previously described methodology, lipid peroxidation was measured as MDA (Mihara and Uchiyama 1978).

According to the manufacturer’s instructions, superoxide dismutase (SOD) activity was estimated colorimetrically in the heart tissues. In this test, SOD prevents the reduction of the nitro blue tetrazolium dye, estimated at a maximum wavelength of 560 nm, and the change in absorbance measured its activity. The colorimetric evaluation of SOD activity was performed following the method designated earlier (Marklund 1985).

As directed by the manufacturer, a colorimetric kit from (Bio Diagnostic, Giza, Egypt, Catalogue No. GP 2524) was used to measure the glutathione peroxidase (GPx) activity. GPx activity was estimated based on the previously described method (Paglia and Valentine 1967).

Nicotinamide adenine dinucleotide phosphate hydrogen (NADPH) is oxidized to NADP + along with GPx activity, which can be measured at a maximum wavelength of 340 nm.

Determination of reduced glutathione (GSH) and total nitrite end product (NO_2_) levels was evaluated via chemically assay according to the methods described by Sedlak and Lindsay (1968) and Montgomery and Dymock (1961), respectively.

Myeloperoxidase (MPO) was estimated according to the method described earlier by Manktelow and Meyer (1986).

According to the manufacturer’s instructions, the NADPH oxidase was determined using an ELISA kit (Catalog No. ELK2600, ELK Biotechnology, Wuhan, China).

According to the manufacturer’s instructions, heme oxygenase 1 (HO-1) was estimated using the sandwich ELISA technique by the kit (Catalog No. ELK1477, ELK Biotechnology, Wuhan, China).

8-Hydroxydeoxyguanosine (8-OHdG) was estimated using the ELISA technique with the kit (Catalog No. E-EL-0028, Elabscience, TX, USA) according to the manufacturer’s instructions.

### Examination of pro-inflammatory mediators in the cardiac tissue

For the determination of the pro-inflammatory cytokines in the cardiac tissue, commercial ELISA kits were used for IL-1β, IL-6, and TNF-α. (Catalog Nos. are ELK1272, ELK1158, and ELK1396), respectively. All of them were purchased from ELK Biotechnology, Wuhan, China. The methodology was performed according to the manufacturer’s instructions.

### Evaluation of cardiotoxicity biomarkers in serum

A colorimetric technique determined the level of alanine aminotransferase (ALT) according to the following reaction:

$$Alanine+\alpha -ketoglutarate\stackrel{ALT}{\to }Pyruvate+Glutamate$$

Then the formed pyruvate is then measured in its derivative form, 2,4-dinitro phenylhydrazone (Bio Diagnostic, Giza, Egypt). Measurement of aspartate aminotransferase (AST) concentration was performed using the international federation of clinical chemistry IFCC method, according to Bergmeyer et al. (1986). cTn-1 was measured in serum using the sandwich-ELISA technique according to the manufacturer’s instructions (Catalog No. E-EL-R1253, Elabscience, TX, USA).

The creatine kinase-myoglobin binding (CK-MB) level was evaluated kinetically using the kit BioMed-CK-MB (Catalog No. CKM107050, Biomed Diagnostics, Inc, OR, USA) according to the manufacturer’s instructions. Kinetic determination of LDH (Catalog No. LDAH 117,025), LDH activity in serum was calculated by measuring the decrease in absorbance per time at 340 nm (Biomed Diagnostics, Inc, OR, USA).

### Estimation of serum electrolytes levels

The amount of sodium (Catalog No. SOD 100,040), potassium (Catalog No. POT 100,040), and calcium (Catalog No. CAL 103,060) was measured by colorimetric assay according to the manufacturer’s instructions (Biomed Diagnostics, Inc, OR, USA).

### Western blotting

Total protein was removed from cardiac tissue after 24 h of treatment with radioimmunoprecipitation assay buffer (RIPA) containing protease inhibitor cocktail (Biospes, China) and quantified using a Bicinchoninic acid (BCA) protein quantification kit (Beyotime Biyuntian Biotechnology Co., Ltd., China). The rats’ heart tissue lysates were loaded onto 7, 10, or 12% SDS–PAGE gels for separation, consistent with the molecular weight of the target proteins, and then transferred onto PVDF membranes (Bio-Rad, Hercules, CA, USA). At room temperature, the membrane was blocked with 5% skim milk in TBST on a shaker for 2 h before its incubation with primary antibodies [toll-like receptor 4 (TRL4, 1:1000, Catalog No. sc-293072, Santa Cruz Biotechnology, Inc.), mTOR (1:1000, Catalog No. sc-517464, Santa Cruz Biotechnology, USA), AMPK (1:1000, Catalog No. sc-74461, Santa Cruz Biotechnology, USA), and nuclear factor kappa B (NF-κB-p65, 1:1000, Catalog No. abx012874, Abbexa, UK), β actin (Catalog No. E-AB-20031, Elabscience, USA)] at 4 °C overnight. TBST was used to wash the membrane for 10 min three times. Afterward, it was incubated with the appropriate horseradish peroxidase-conjugated secondary antibody against IgG for rabbits and mice. The membrane was then rewashed with TBST three times for 10 min. The target protein was exposed to an ECL chemiluminescent system (Tanon-5200Multi, Shanghai, China). The target protein band value was then calculated using ImageJ 1.8.0 software (NIH).

### Histopathological examination

#### Light microscopy

Myocardial injuries in rats were evaluated by the scoring system, according to Atkinson et al. (2010). The findings were categorized into the following grades to compose a range of histological myocardial injuries considering scores from 0 to 3 for the categories: edema, myocardial hemorrhage, inflammation, and necrosis. The score was assigned to (0) absence of damage, (1) slight degree of damage, (2) moderate damage, and (3) maximum degree of damage. This methodology was used to evaluate the heart histopathological (HP) index.

#### Immunohistochemical examination

Caspase-3 as an apoptotic marker was evaluated using the methodology of Hsu et al. (1981). According to Rashad and colleagues, the anti-apoptotic B-cell lymphoma2 (BCL2) was estimated (Rashad et al. 2022).

The immunohistochemical observations were evaluated using image analysis (area percentage). Sample sections were stained with anti-caspase-3 and anti-BCL2 antibodies and then analyzed by a digital Leica Quin 500Â image analysis system (Leica Microsystems, Switzerland) at the Faculty of Dentistry, Cairo University. The image analyzer was automatically calibrated to translate pixels into area units (μm2). Caspase-3 and BCL2 immunostaining was presented as a percentage of the total area in a standard measuring frame over ten independent fields from different slides in each group at × 400 magnification. Regardless of the intensity, all areas with positive immunohistochemical staining were assessed. All results were presented as mean ± standard deviation (SD).

### Statistical analysis

All results were presented as mean and standard deviation. One-way ANOVA was performed, and Tukey’s post hoc test was carried out for data analysis and comparisons between groups. All analyses were carried out using GraphPad Prism version 6.04. A *P*-value less than 0.05 was considered statistically significant.

## Results

### The antioxidant effect of β-sitosterol or trimetazidine on potassium dichromate-induced cardiotoxicity in a rat model

**Fig. 1:**
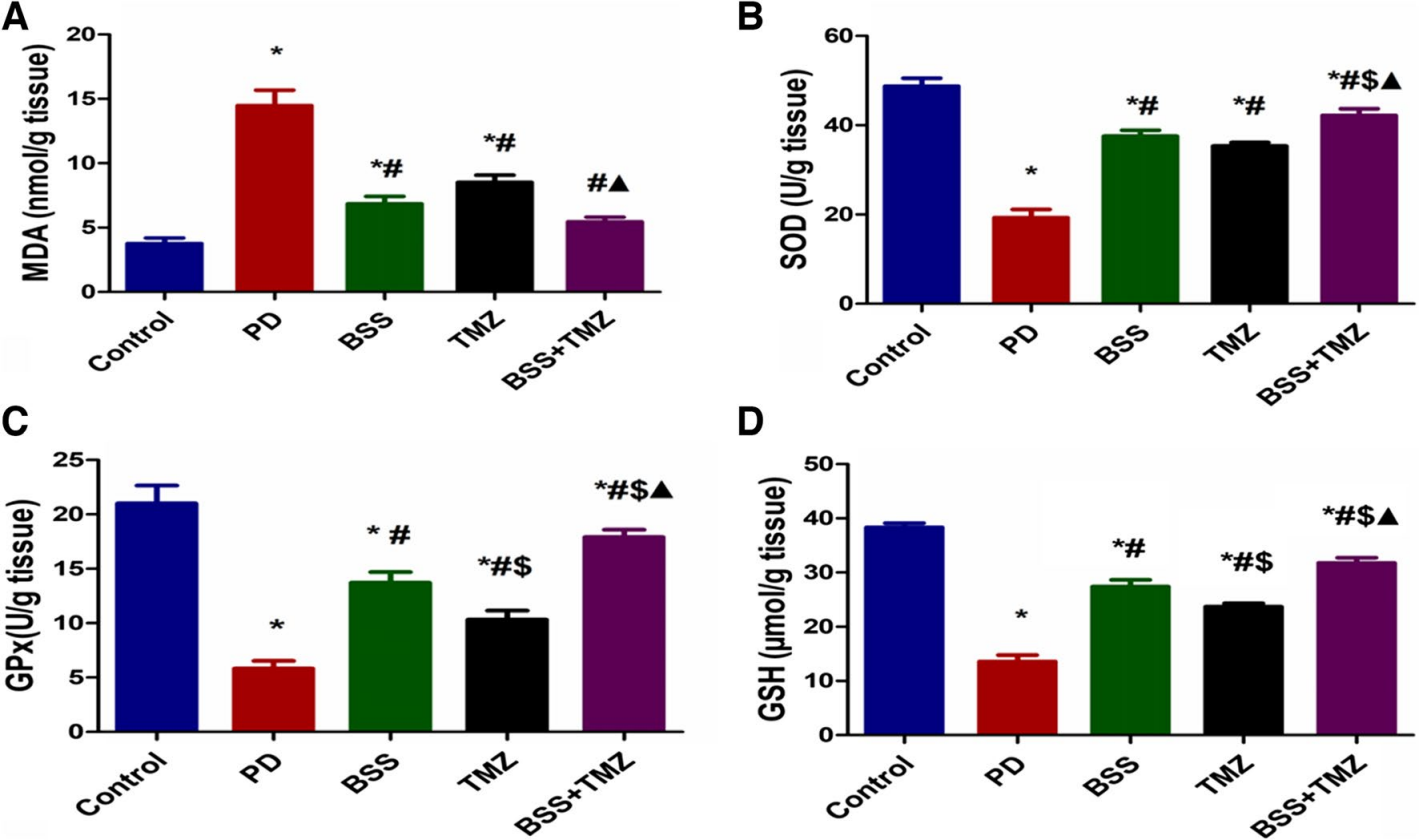
Effects of administering BSS and TMZ on levels of oxidative stress markers: **A** MDA (nmol/g tissue), **B** SOD (U/g tissue), **C** GPx (U/g tissue), and **D** GSH (mmol/mg tissue) in PD cardiotoxicity induced in rats. Data were expressed as mean ± SD and were examined using one-way ANOVA followed by Tukey’s post hoc test. * Significantly differs from the control group; #significantly differs from the PD group; ^$^ significantly differ from BSS group; ^▲^significantly differs from TMZ group at *p* < 0.05. *n* = 6. MDA, malondialdehyde; SOD, super oxide dismutase; GPx, glutathione peroxidase; GSH, glutathione; PD, potassium dichromate; BSS, β-sitosterol; TMZ, trimetazidine

The current results revealed that both BSS and TMZ had succeeded individually in reducing the cellular polyunsaturated fatty acid peroxidation by lowering the level of MDA in rats’ cardiac homogenates. However, combined, they returned MDA to normal levels, as shown in Fig. 1A. The lowest levels of SOD were found in PD-treated rat samples, while BSS and TMZ treatment could significantly enhance SOD levels. Moreover, the combination of BSS and TMZ showed the best results compared to each of them solely (Fig. 1B).

**Fig. 2:**
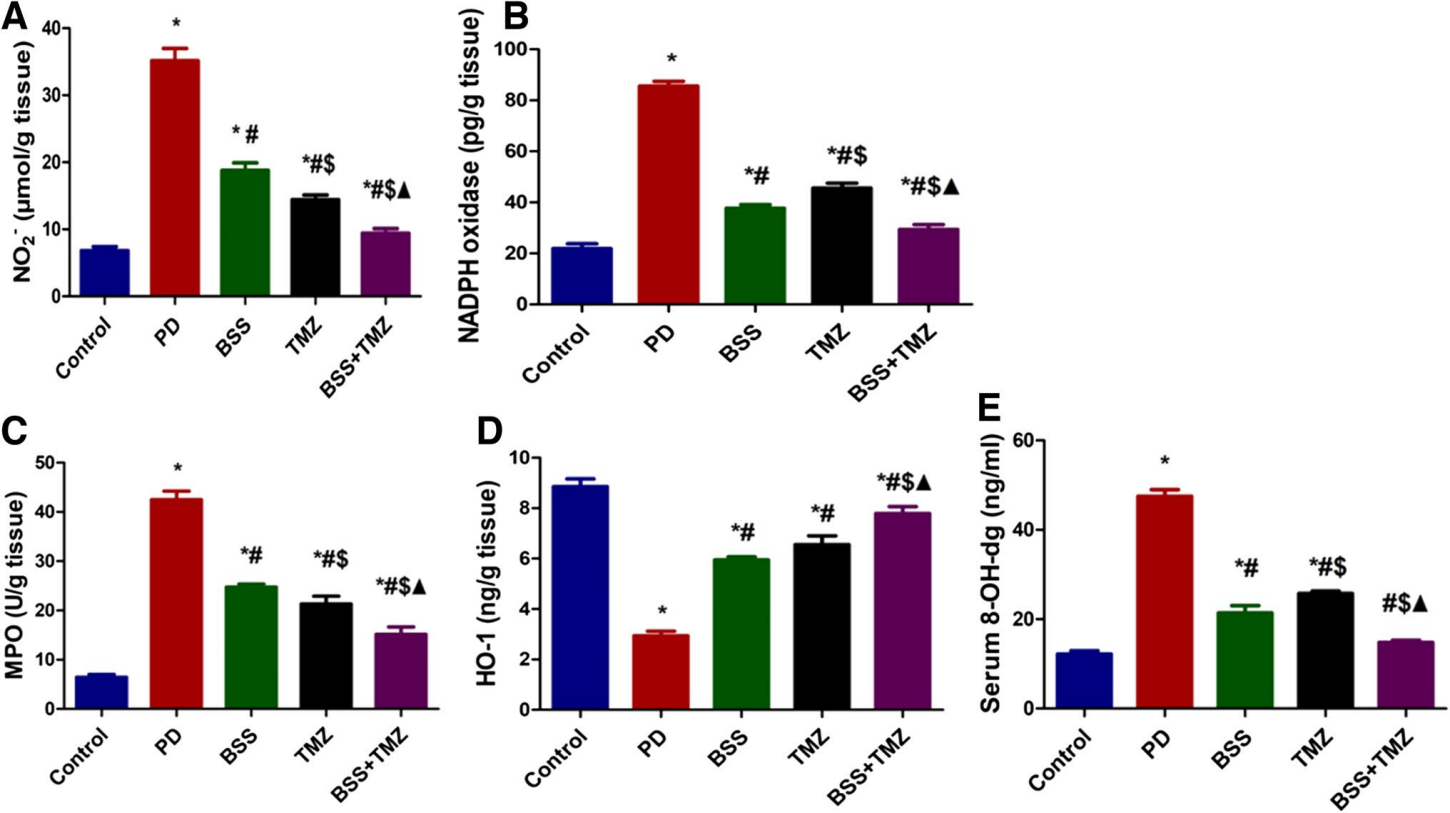
Effects of administering BSS and TMZ on levels of oxidative stress markers:** A** NO_2_^−^, (µmol/g tissue), **B** NADPH oxidase (pg/g tissue), **C** MPO (U/g tissue), **D** HO-1 (ng/g tissue), and and **E** 8-OH-dg (ng/mL) in PD cardiotoxicity induced in rats. Data were expressed as mean ± SD and were examined using one-way ANOVA followed by Tukey’s post hoc test. * Significantly differs from the control group; #significantly differs from the PD group; ^$^ significantly differ from the BSS group; ^▲^significantly differs from TMZ group at *p* < 0.05. *n* = 6. NO_2_^−^, nitric oxide end-product; NADPH oxidase, nicotinamide adenine dinucleotide phosphate oxidase; MPO, myeloperoxidase enzyme; HO-1, heme oxygenase-1; 8-OH-dg, 8-hydroxy-2-deoxyguanosine; PD, potassium dichromate; BSS, β-sitosterol; TMZ, trimetazidine

The highest levels of glutathione and glutathione peroxidase were found in the control group. The PD administration managed to inhibit the GPx and GSH levels severely. BSS and TMZ combination showed the best results in enhancing both GPx and GSH levels to return them near normal levels, as demonstrated in Fig. 1C and 1D. The current study reported significantly higher NO_2_^−^ levels in rats of PD-treated group. BSS or TMZ treatment downregulates the NO_2_^−^ levels to be closer to normal levels. Interestingly, the combination treatment succeeded in normalizing the levels of NO_2_^−^ as shown in (Fig. 2A).

The NADPH oxidase was at its highest levels in the PD group rats. Administering BSS or TMZ or both of them managed to downregulate NADPH oxidase levels; however, the combination treatment showed closer levels to normal ones (Fig. 2B).

Additionally, the myeloperoxidase enzyme showed its highest levels in rats of PD groups, while the group administered BSS and TMZ combination was the group showing the minor MPO levels of the tested groups and the nearest one to normal controls (Fig. 2C).

Figure 2D shows that HO-1 protein was at its lowest level in the PD group. Administering BSS or TMZ significantly elevated HO-1 levels; however, the combination BSS + TMZ showed the highest levels among the tested group (Fig. 2D)

One of the biomarkers of the oxidative damage of DNA is 8-hydroxy-2-deoxyguanosine. The present findings reported that the highest levels of 8-OH dg were found in the PD group of rats. Administering BSS or TMZ significantly reduces these serum levels. The combination BSS + TMZ group recorded the lowest levels of 8-OH dg among the tested groups, as shown in Fig. 2E.

### The anti-inflammatory effect of β-sitosterol or trimetazidine on potassium dichromate-induced cardiotoxicity in a rat model

**Fig. 3:**
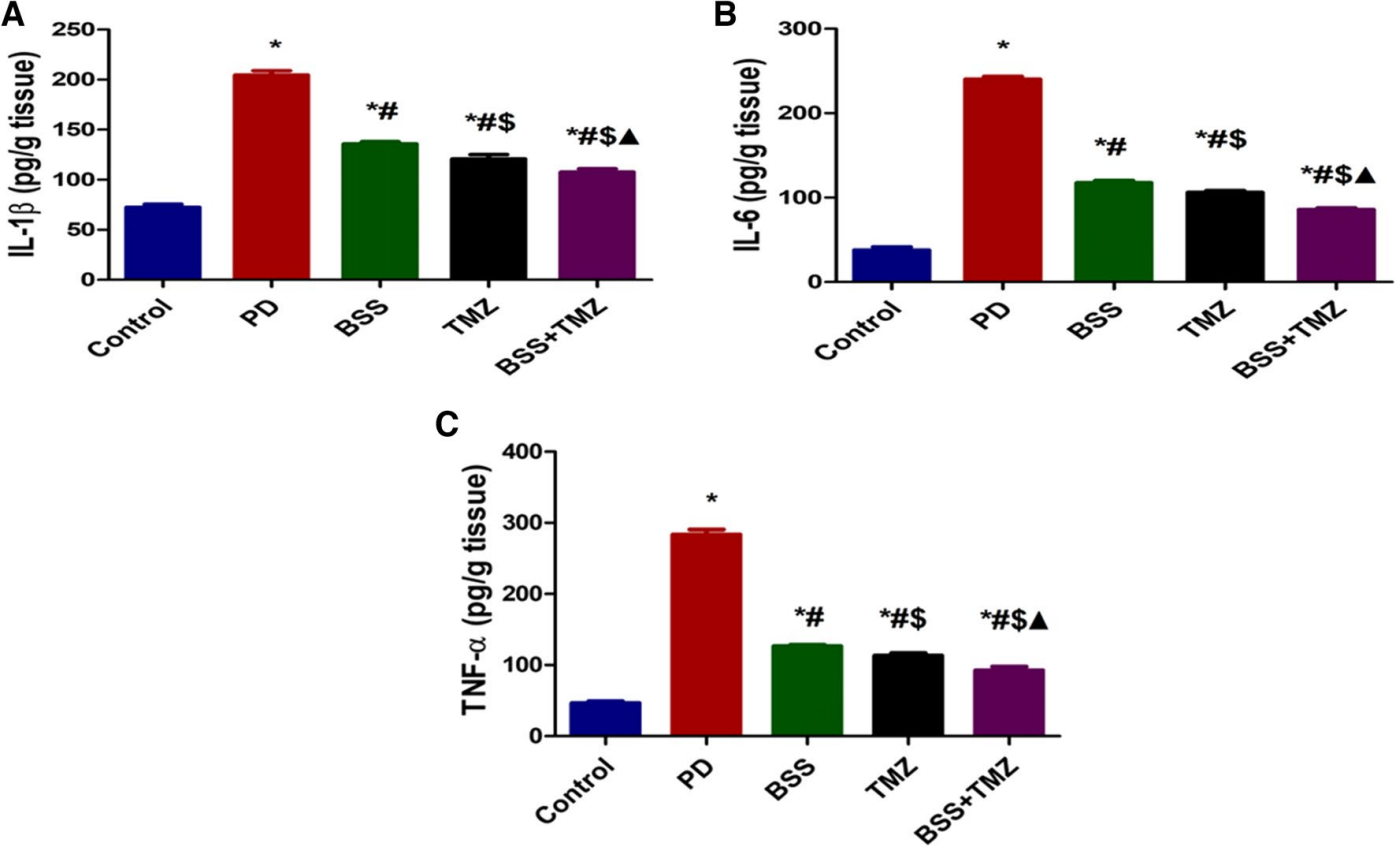
Effects of administering BSS and TMZ on levels of inflammatory markers: **A** IL-1β (pg/g tissue), **B** IL-6 (pg/g tissue), and **C** TNF-α (pg/g tissue) in PD cardiotoxicity induced in rats. Data are expressed as mean ± SD and were examined using one-way ANOVA followed by Tukey's post hoc test. * Significantly differs from the control group; #significantly differs from the PD group; ^$^ significantly differs from BSS group; ^▲^ significantly differs from the TMZ group at *p* < 0.05. *n* = 6. IL-1β, interleukin-β; IL-6, interleukin-6; TNF-α, tumor necrosis factor-α; PD, potassium dichromate; BSS, β-sitosterol; TMZ, trimetazidine

The current study highlighted the anti-inflammatory effect of both tested drugs alone or combined through several biomarkers. The highly elevated IL-1β levels found in tissue homogenate samples of the PD group were significantly inhibited when BSS or TMZ were used. However, their combination showed the lowest levels among tested groups, as shown in Fig. 3A.

Similarly, BSS and TMZ administration reduced IL-6 levels compared to the PD group, but the combination showed the most promising results (Fig. 3B).

Tumor necrosis factor-α showed its most elevated levels in the PD group. BSS- and TMZ-treated groups showed significantly inhibited TNF-α levels, while their combination was more beneficial in reducing levels of TNF-α (Fig. 3C).

### The effect of β-sitosterol or trimetazidine on levels of cardiotoxicity biomarkers in potassium dichromate-induced cardiotoxicity in a rat model

**Fig. 4:**
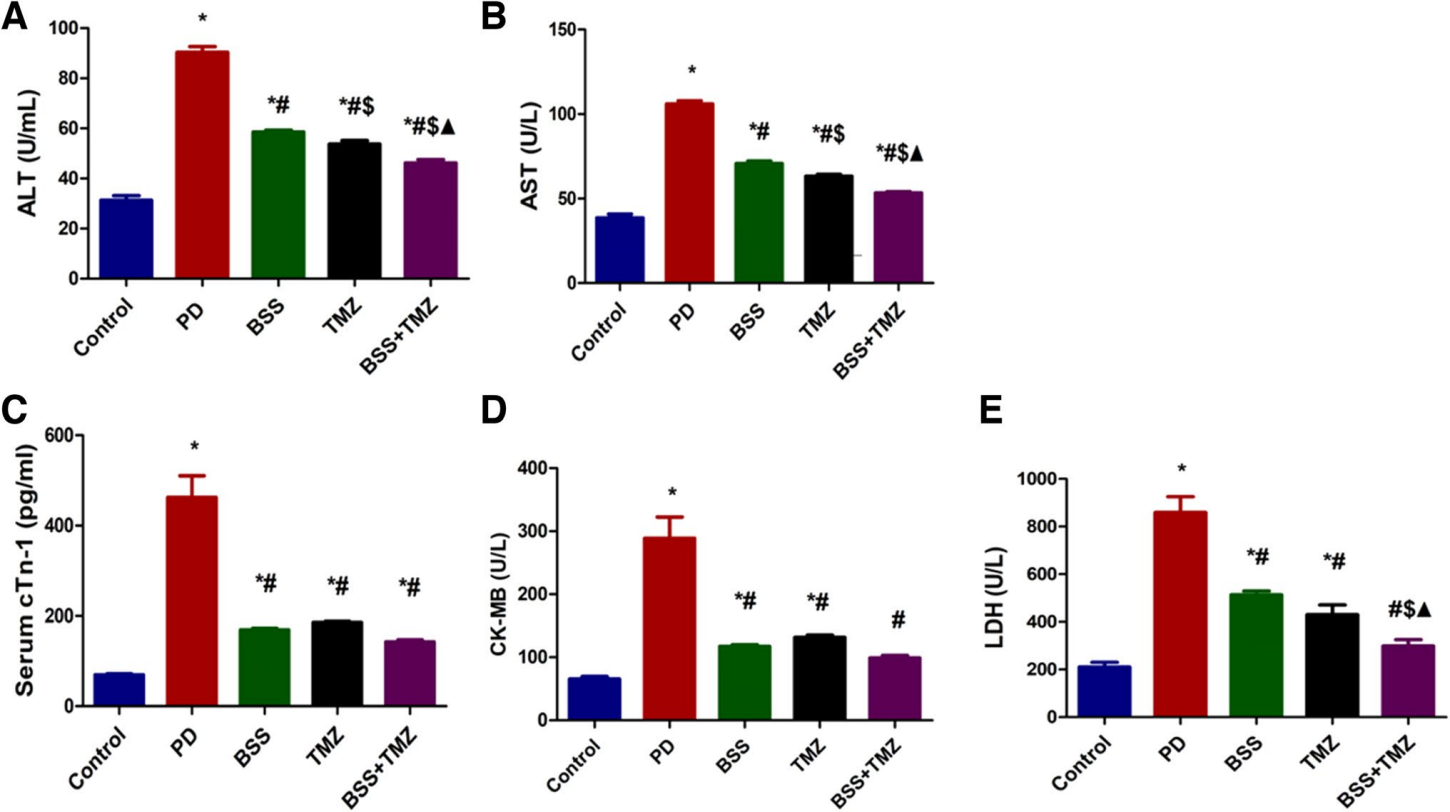
Effects of administering BSS and TMZ on serum levels of several enzymes:** A** ALT, (U/mL), **B** AST (U/L), **C** cTn-1 (pg/mL), **D** CK-MB (U/L), and **E** LDH (U/L) in PD cardiotoxicity induced in rats. Data are expressed as mean ± SD and were examined using one-way ANOVA followed by Tukey’s post hoc test. * Significantly differs from the control group; #significantly differs from the PD group; ^$^ significantly differ from BSS group; ^▲^ significantly differs from TMZ group at *p* < 0.05. *n* = 6. ALT, alanine transaminase; AST, aspartate transaminase; cTn-1, cardiac troponin; CK-MB, creatine kinase-MB; LDH, lactate dehydrogenase; PD, potassium dichromate; BSS, β-sitosterol; TMZ, trimetazidine

The PD group demonstrated the most elevated level of ALT and AST enzymes. After adding BSS or TMZ, the enzyme levels decreased significantly. However, the combination group showed the lowest levels of enzymes among the tested groups (Fig. 4A and B).

Figure 4 reveals the elevated levels of cardiac enzyme troponin and CK-MB in the PD group of rats to the highest most significant levels; while adding BSS or TMZ or their combination, the cardiac enzyme levels were significantly inhibited. Also, the BSS + TMZ managed to return the cardiac troponin (cTn-1) level to normal (Fig. 4C and D). The administration of BSS or TMZ reduced the level of lactate dehydrogenase enzyme in the serum of the rats compared to the extremely high LDH levels in the serum of PD-treated rats. However, their combination revealed the lowest LDH serum levels among the tested groups (Fig. 4E).

### The effect of β-sitosterol or trimetazidine on levels of serum electrolytes in potassium dichromate-induced cardiotoxicity in a rat model

**Fig. 5:**
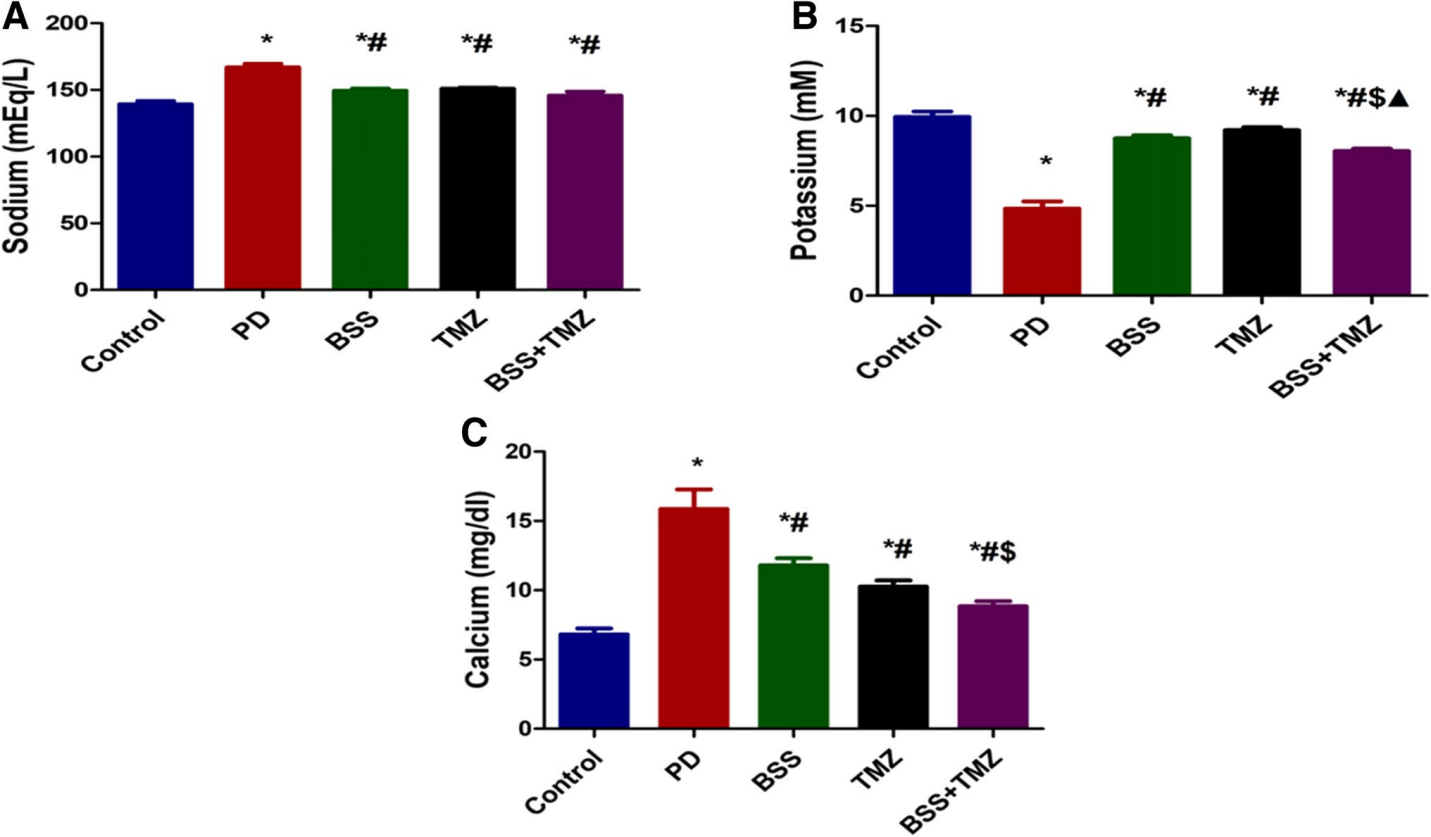
Effects of administering BSS and TMZ on serum levels of several electrolytes:** A** sodium (mEq/L), **B** potassium (mM), **C** calcium (mg/dL), and **D** CK-MB (U/L) in PD cardiotoxicity induced in rats. Data are expressed as mean ± SD and was examined using one-way ANOVA followed by Tukey’s post hoc test. * Significantly differs from the control group; # significantly differs from the PD group; ^$^ significantly differ from BSS group; ^▲^ significantly differs from TMZ group at *p* < 0.05. *n* = 6. PD, potassium dichromate; BSS, β-sitosterol; TMZ, trimetazidine

The current findings revealed that the sodium serum levels of rats in the PD group were significantly higher than normal. After administration of BSS or TMZ or their combination, sodium levels were inhibited significantly compared to the PD group (Fig. 5A).

The potassium level in the PD group was downregulated than the normal level. However, the three other tested groups showed significantly higher potassium levels than the PD group, as shown in Fig. 5 B. The highest calcium levels were shown in the PD group. In the BSS, TMZ, and BSS + TMZ groups, the calcium levels were inhibited from becoming closer to normal (Fig. 5C).

### The effect of β-sitosterol or trimetazidine on protein expression of TLR4, mTOR, NF-κB-p65, and AMPK in heart tissue in potassium dichromate-induced cardiotoxicity in a rat model

**Fig. 6:**
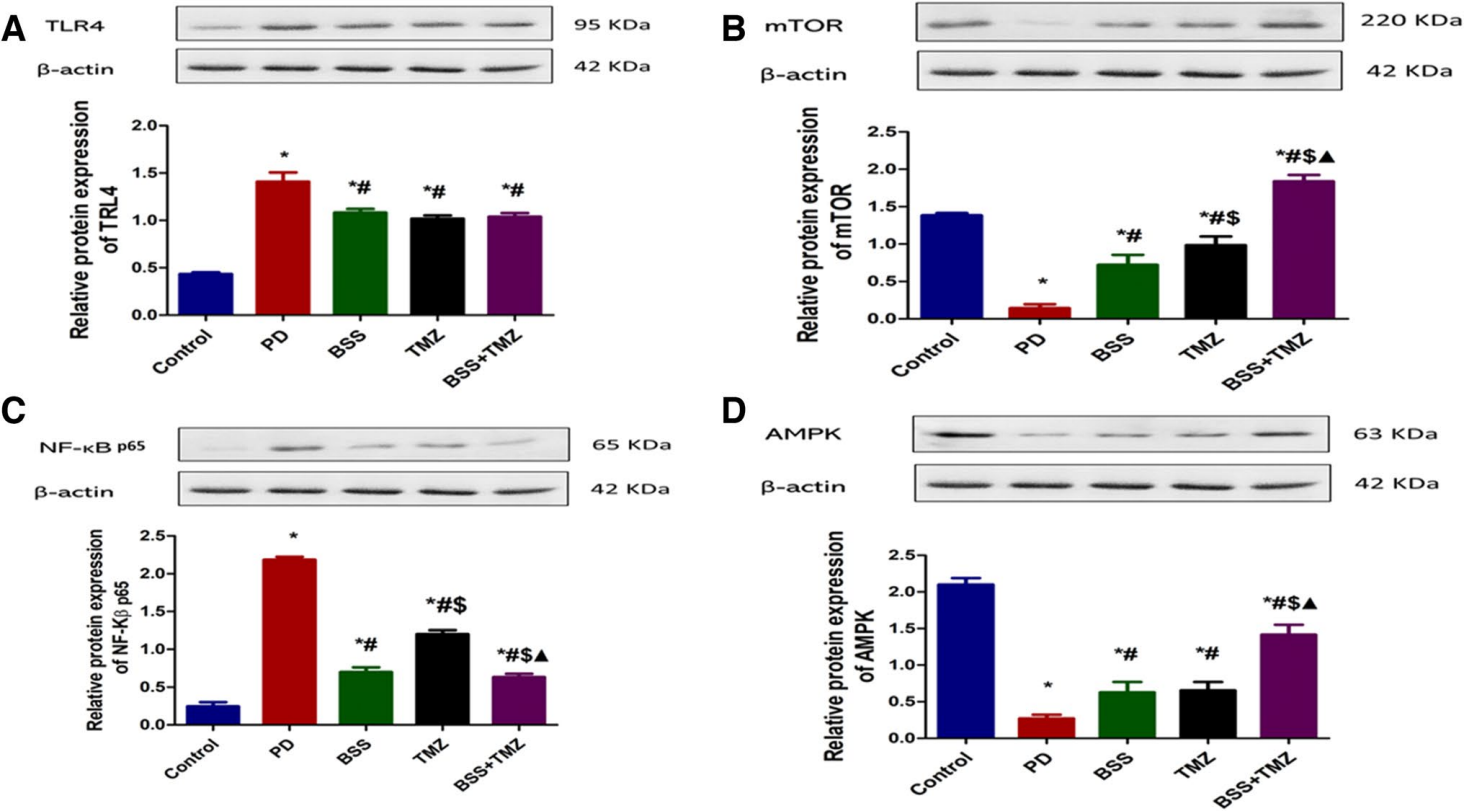
Effects of administering BSS and TMZ on relative protein expression of **A** TRL4, **B** mTOR, **C** NF-κB p65, and **D** AMPK in PD cardiotoxicity induced in rats. Data are expressed as mean ± SD and was examined using one-way ANOVA followed by Tukey's post hoc test. * Significantly differs from the control group; # significantly differs from the PD group, ^$^ significantly differ from BSS group; ^▲^ significantly differs from TMZ group at *p* < 0.05. TLR4, toll-like receptor 4; mTOR, mammalian target of rapamycin; NFkB, nuclear factor kappa B; AMPK, AMP-activated protein kinase; PD, potassium dichromate; BSS, β-sitosterol; TMZ, trimetazidine. *n* = 6

Figure 6 highlights several protein expression levels, representing major pathways. The current findings demonstrate that the PD group showed the highest TLR4 and NF-kB p65 protein expression levels. However, the three tested treatments showed almost the same reduction in TLR4 expression. TMZ, BSS, and BSS + TMZ groups showed the same reduction levels in NF-kB p65 protein expression (Fig. 6 A and C). Furthermore, the PD group’s obviously low mTOR and AMPK protein level expression was best normalized when the combination treatment (BSS + TMZ) was used (Fig. 6B and D).

### The effect of β-sitosterol or trimetazidine on the light microscopic observations

**Fig. 7:**
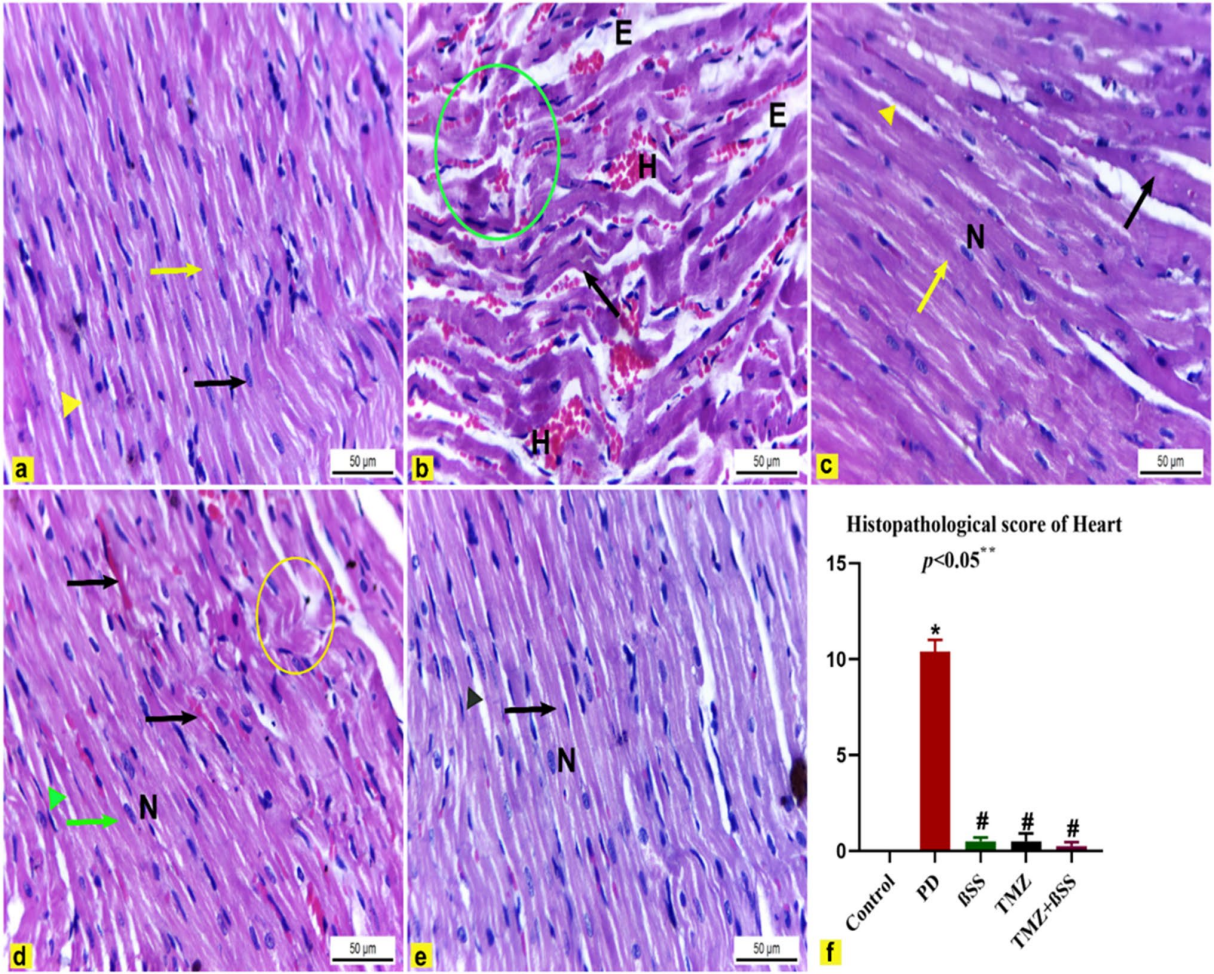
**a**, **f** Heart sections of male albino rats. H&E stain × 400. **a** Control rats showed normal elongated striated, cardiac myofibers (yellow arrow), and had an oval central nuclei (black arrow) and interstices between them (yellow arrowhead). **b** PD-intoxicated rats had degenerative alteration such as wavy-shaped myofibers with unclear striation (black arrow), hemorrhage (H), edema (E), and necrotic cardiac myofibers (green circle). **c** PD-treated rats with BSS administration showed a marked protective effect against PD toxicity in the form of nearly normal cardiac myofibers that appeared elongated branched (yellow arrow) with central oval nuclei (N) and interstices between them (yellow arrow head). Except for a few myofibers appeared necrotic (black arrow). **d** PD-treated rats with TMZ administration revealed a decrease in the hemorrhage (black arrow), few myofibers appeared wavy in shape (yellow circle), and other cardiac myofibers appeared nearly normal elongated branched (green arrow). They contained an oval central nucleus (N) with interstices (green arrowhead) between them. **e** PD-treated rats administered with both BSS and TMZ showed nearly normal elongated branched striated, cardiac myofibers (black arrow) containing oval central nuclei (N) with interstices (black arrowhead) between them. **f** Heart histopathological score. The results were expressed as mean ± SD. * Significantly different from the control group; ^#^ significantly different from the PD group. *p* value ≤ 0.05

H&E-stained heart sections of control rats showed normal histological architecture of cardiac muscles, which appeared elongated branched and cross-striated myofibers with oval central nuclei. Between myofibers, narrow slit-like interstices contained loose CT and blood capillaries (Fig. 7A). Contrariwise, heart sections obtained from PD-intoxicated rats revealed various degenerative alterations of cardiac muscles compared with the control group like wavy-shaped myofibers with unclear striation, some myofibers appeared necrotic, hemorrhage, and edema between cardiac myofibers (Fig. 7B). Conversely, administration of BSS to PD-intoxicated rats exhibited a marked protective effect against the cardiac muscular degenerative changes compared to the PD group induced by PD, evidenced by restoring the normal structure of cardiac myofibers that appeared elongated branched and striated with central oval nuclei. There was no hemorrhage or edema in the interstices between the myofibers. Few myofibers were still necrotic (Fig. 7C). However, TMZ administration to PD-intoxicated rats revealed a marked decrease in the histopathological changes of cardiac muscles in comparison with the PD group in the form of a decrease the hemorrhage, few myofibers appeared wavy in shape, and other cardiac myofibers appeared nearly normal elongated branched contained oval central nucleus with interstices between them (Fig. 7D). Finally, the co-administration of both BSS and TMZ to PD-treated rats showed significant recovery of cardiac muscular degenerative alterations compared to the PD group, which was proved by the nearly normal appearance of elongated branched cardiac myofibers that had oval central nuclei with interstices between them (Fig. 7E).

### The effect of β-sitosterol or trimetazidine on histopathological score observations

The cardiac muscle histopathological score was significantly higher in rats treated with PD than in control rats. But the score was significantly (*P* ≤ 0.05) lower in PD-treated rats which received BSS, TMZ, and BSS + TMZ than in rats treated with PD alone (Fig. 7F).

### The effect of β-sitosterol or trimetazidine on immunohistochemical investigations

**Fig. 8:**
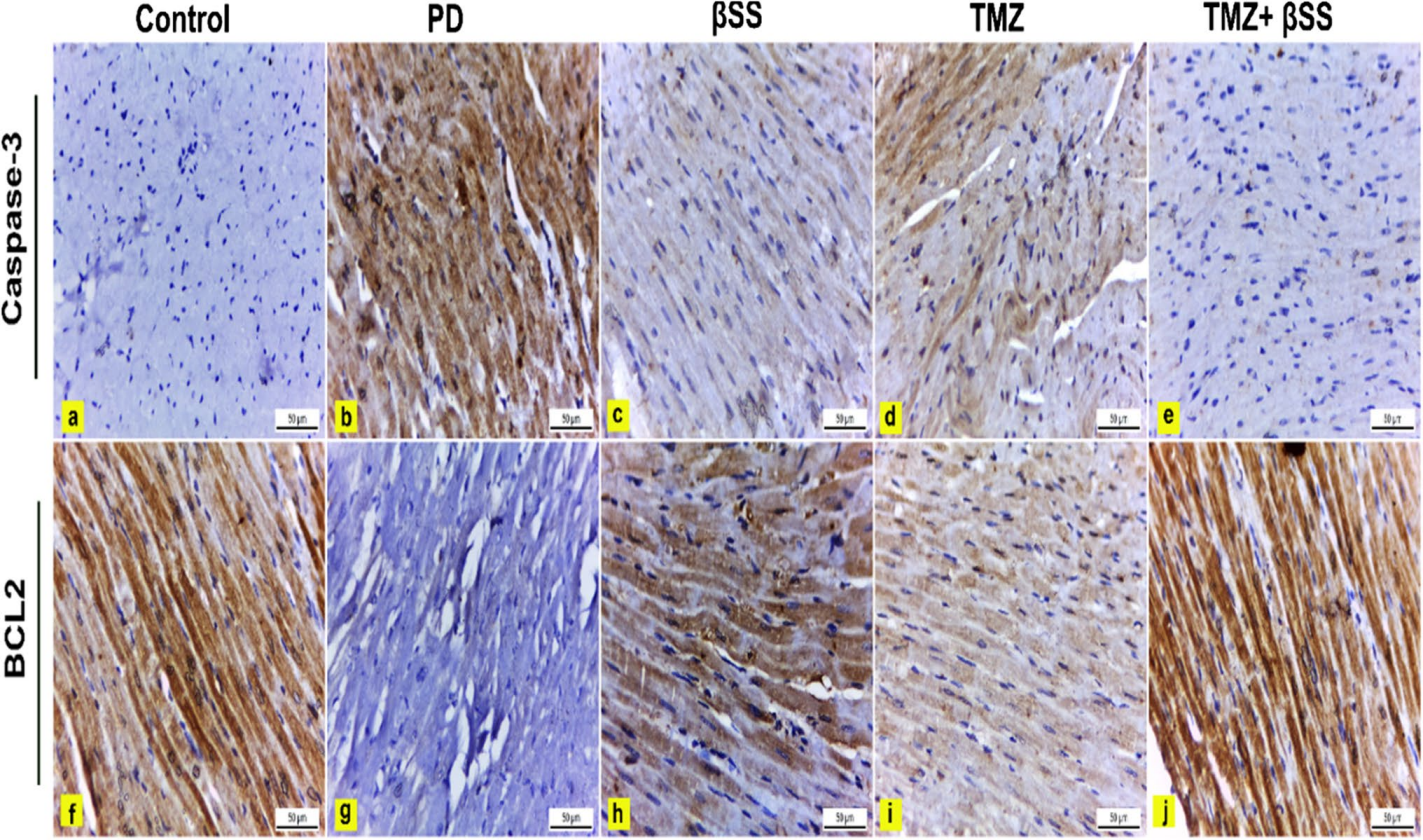
Immunohistochemically caspase-3 (**a**, **e**) and BCL2 (**f**, **j**) stained heart sections, × 400. **a** Control rats had negligible caspase-3 immunoexpression in cardiac myofibers. **b** PD-treated rats showed strong positive caspas-3 expression in cardiac myofiber compared to the control group. **c** PD-treated rats with BSS administration revealed mild immunoexpression versus the PD group. **d** PD-treated rats with TMZ administration showed mild immunoexpression versus the PD group. **e** PD-treated rats with BSS and TMZ exhibited negligible caspase-3 immunoreactivity versus control, PD, BSS, and TMZ groups. **f** Control rats had strong positive BCL2 immunoexpression in cardiac myofibers. **g** PD-intoxicated rats showed negligible BCL2 immunoreaction versus the control group. **h** PD-treated rats with BSS administration revealed moderate BCL2 immunoreactivity compared to the PD group. **i** PD-treated rats with TMZ administration showed mild BCL2 immunoreaction versus the PD group. **j** PD-treated rats with BSS and TMZ revealed strong positive BCL2 expression versus the PD group. BCL2, B-cell lymphoma 2

**Fig. 9:**
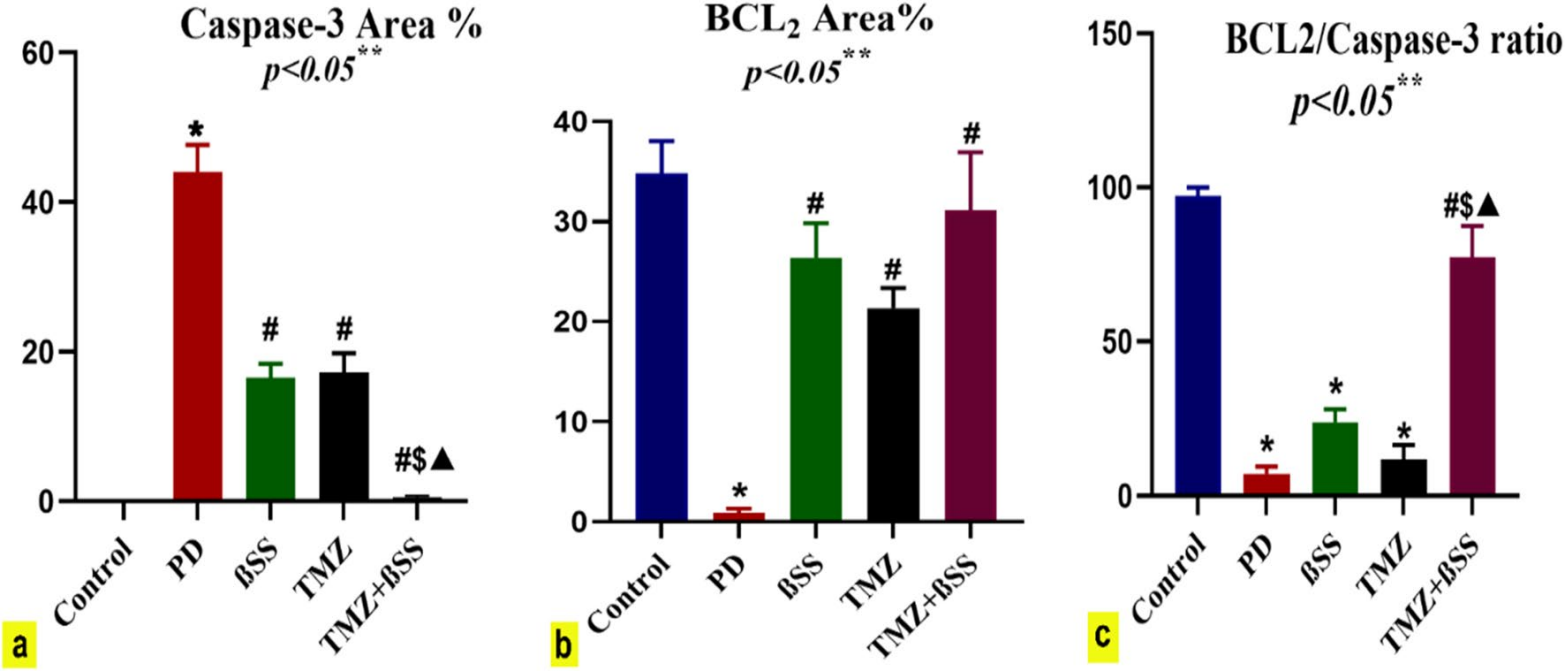
**a**–**c** The effect of PD, PD + BSS, PD + TMZ, and PD + BSS + TMZ on the percent area covered by caspase-3, BCL2, and BCL2/caspase-3 ratio positive immunoreactive cells, respectively, within the heart of rat. The results were expressed as mean ± SD. * Significantly different from the control group; # significantly different from the PD group; $ significantly different from the BSS group; ▲significantly different from the TMZ group. *p* value ≤ 0.05

Immunohistochemical examination of caspase-3 stained heart sections of control rats showed negative immunoexpression in cardiac muscles (Fig. 8a). However, cardiac muscles of PD-intoxicated rats revealed significantly strong positive caspase-3 immunoreaction by 43.9% compared to the control group (Fig. 8 b and c). Contrariwise, administration of BSS and TMZ to PD-treated rats revealed mild caspase-3 immunoexpression in cardiac myofibers that significantly reduced by 16.5% and 17.2%, respectively, compared to the PD group (Fig. 8c, d, and 9a). Moreover, administration of both BSS and TMZ to PD-treated rats exhibited negligible caspase-3 immunoreactivity that significantly decreased by 0.5% compared to PD, BSS, and TMZ groups (Figs. 8e and 9a).

Immunohistochemical examination of BCL2-stained heart sections of control rats revealed strong positive immunoreaction in cardiac muscles (Fig. 8f). On the contrary, cardiac muscles of PD-intoxicated rats showed negligible BCL2 immunoexpression that significantly reduced by 0.8% compared to the control group (Figs. 8g and 9b). However, administration of BSS to PD-treated rats exhibited moderate BCL2 immunoreactivity in cardiac muscles that significantly increased by 26.3% compared to the PD group (Figs. 8h and 9b). Moreover, administration of TMZ to PD-treated rats exhibited mild BCL2 immunoreactivity in cardiac muscles that significantly increased by 21.3% versus the PD group (Figs. 8i and 9b). Finally, administering BSS and TMZ to PD-intoxicated rats showed strong positive BCL2 immunoexpression in cardiac muscles that significantly increased by 31.3% versus PD rats (Figs. 8j and 9b).

Moreover, BCL2/caspase-3 ratio revealed a significant decrease (*P* ≤ 0.05) in PD, BSS, and TMZ groups versus the control group. However, BCL2/caspase-3 ratio showed a significant increase (*P* ≤ 0.05) in PD-treated rats administered both BSS and TMZ compared to PD, BSS, and TMZ groups (Fig. 9c).

## Discussion

The current study’s authors attempted to investigate the potential impact of adding either BSS or TMZ or their combination on the cardiotoxicity induced by PD. Many people’s lives could be in danger from occupational or environmental exposure to PD, now recognized as an occupational carcinogen. The damaging effects of PD on various body organs, including the kidneys, liver, and heart, were highlighted in several reports. To prevent this kind of toxicity, it is crucial to understand the mechanism underlying PD-induced cardiotoxicity and the various agents that can be used for prevention. PD-induced cardiotoxicity, its mechanism of action, potential protective agents, and the mechanism of this protection were the main topics of our study.

The current study proved that PD exposure causes various pathological and biochemical changes. The pathophysiological alterations, which included cardiotoxicity and elevated levels of enzymes related to cardiotoxicity, were consistent with earlier studies (Yang et al. 2021; Soudani et al. 2011). These studies covered the various PD-related impairments produced by pro-inflammatory pathways and oxidative stress in several vital organs. On the other hand, the administration of the BSS + TMZ combination completely undid all the abovementioned changes.

The detrimental changes in cardiac tissue, such as degeneration and cardiac cell apoptosis, supported the PD-induced cardiac deficits. According to several studies, PD can damage organs and cause nephrotoxicity, cardiotoxicity, and hepatotoxicity by producing ROS or inducing mitochondrial damage, prompting cell necrosis or apoptosis. Through the overproduction of the lipid peroxidation marker MDA and the decreased activity of several antioxidants, including GPx, GSH, and SOD, oxidative stress has been highlighted as one of the primary factors contributing to PD-related organ damage and degeneration (Soudani et al. 2011; Yang et al. 2021).

By catalyzing the dismutation of the superoxide anion into oxygen and hydrogen peroxide (H_2_O_2_), which GPx then metabolizes, SOD is recognized as the first member of the system of antioxidant defense (Zhen et al. 2016; Hamdan et al. 2022). Additionally, MDA is increasingly known as the most significant byproduct of the peroxidation of membrane lipids, making it a useful indirect marker of the degree of cell damage (Senn et al. 2017). In the current study, PD intoxication increased MDA, NO_2_^−^, and MPO levels and significantly declined the antioxidant enzyme activities of GPx, GSH, and SOD.

Interestingly, the administration of BSS, TMZ, or both significantly reduced the production of MDA and MPO in the cardiac tissue and, as a result, downregulated the percentage of apoptosis. Caspase-3 is a primary proapoptotic mediator and marker of irreversible apoptosis (Khalifa et al. 2017). In the present study, immunohistochemical examination of heart sections of PD-intoxicated rats revealed strong positive caspase-3 expression in cardiac myofibers compared to control rats. Contrariwise, the expression of the anti-apoptotic BCL2 marker in cardiac myofibers was significantly reduced in PD-intoxicated rats. These observations indicated the apoptotic effect of PD on cardiac muscles.

In contrast, administration of BSS and TMZ individually to PD-treated rats resulted in a significant reduction of caspase-3 and elevation of BCL2 immunoexpression in cardiac myofibers compared to the PD group. Moreover, the co-administration of both BSS and TMZ to PD-treated rats resulted in negligible caspase-3 and strong positive BCL2 immunoreaction in cardiac myofibers compared to the PD group. These findings postulate the anti-apoptotic role of both BSS and TMZ, used individually or in combination. Still, to a greater extent, they are more potent against PD when used in combination. This suggests that administering BSS + TMZ can potentially prevent diseases associated with cardiotoxicity. These outcomes were also consistent with the findings of the DPPH assay and demonstrated the potential modulating effect of BSS and TMZ on the antioxidant pathway.

Several intracellular signal transduction pathways are activated and started when toxic products engage TLRs. One of the most notable of these is the one that activates NF-κB-p65. The innate host defense system’s most crucial component is TLR-induced NF-κB-p65 activation. That was in agreement with our findings which confirmed the upregulation of both TLR-4 and NF-κB-p65, indicating the pro-inflammatory effect of PD. Preventive therapies succeeded in downregulating both proteins close to normal values (Zhang and Ghosh 2001; Zaafar et al. 2022).

Furthermore, NF-κB-p65, which generates cytokines that promote inflammation and cytotoxic genes, is known to increase ROS production (Hu et al. 2020). In the present study, PD-intoxicated rats exhibited a strong expression of NF-κB-p65. This outcome is consistent with a study published earlier (Sahu et al. 2014). The enzyme HO-1 is thought to respond to stress and has a primary antioxidant and anti-inflammatory function. It can catabolize heme into iron, carbon monoxide, and biliverdin. As a result, it is crucial for cardiac protection. Therefore, HO-1 downregulation might harm the cardiac tissue. This was consistent with recent findings that showed rats exposed to PD had the best upregulation of HO-1 when BSS and TMZ were used as a protection mechanism. These results could be related to the antioxidant effect of BSS and TMZ and the ability to regulate several pathways, including NF-κB-p65, Nrf2, and HO-1.

The expression of HO-1 has substantial protective effects against ROS-induced oxidative damage both in vitro and in vivo (Datla et al. 2007). Taille and colleagues showed that HO-1 expression inhibited NADPH oxidase in macrophages by decreasing the heme availability and Nox2 protein abundance (Taillé et al. 2004). Our findings showed that HO-1 modulated the NADPH oxidase activity and SOD production in vivo. The inhibitory effect of HO-1 was observed in the heart tissue of PD-intoxicated rats, in which NADPH oxidase activation is a major source of oxidative stress. This is agreed with several studies showing that HO-1 expression suppresses NADPH oxidase-dependent superoxide production (Jiang et al. 2006; Wilcox 2005). This could be a key mechanism underlying how HO-1 protects the cardiovascular system.

Nitrite anion is a reactive nitrogen species associated with tissue injury or disease development. As was previously demonstrated, these species are also produced when exposed to exogenous insults like environmental pollutants (Griendling et al. 2016). Therefore, the high insult on cardiac tissue is linked to the highest nitrite ions found in the PD group. The combination group’s lowest nitrite ion level is evidence of its potent cardiac tissue protection.

The most critical biomarkers for oxidative DNA damage are the guanine and deoxyguanosine oxidation products produced from oxidative stress by ROS or RNS. Excessive 8-hydroxy-2-deoxyguanosine levels may indicate a recent insult to an organ. These oxidative DNA damages may result in genomic instability, persistent inflammation, carcinogenicity, or cell death by causing changes or mutations within the cell’s genomic material (Chiorcea-Paquim 2022). Our research was consistent with these findings as the PD group had the highest levels of 8-oh-dg. Combination therapy was the most effective treatment for returning those levels to normal ranges.

In this study, we highlighted that the combination of BSS + TMZ significantly increased AMPK phosphorylation in the PD-induced cardiotoxicity model. Our results align with Ye and colleagues, who reported the cardioprotective effect of dapagliflozin via mechanism depending on AMPK (Ye et al. 2017).

The crosstalk between AMPK and NF-κB-p65 signaling has also been highlighted previously. AMPK signaling was demonstrated to suppress the NF-κB-p65 signaling pathway and subsequent expression of pro-inflammatory biomarkers (Huang et al. 2015). Chen and colleagues revealed that AMPK activation downregulated the NF-κB-p65 signaling cascade in H9c2 cells (Chen et al. 2018). In the same line, the activation of AMPK inhibited the translocation of NF-κB-p65 from the cytosol to the nucleus in activated macrophages of inflamed skin tissues (Xiang et al. 2019).

Several studies have highlighted the critical role of intact mTOR signaling in cell survival and function and evidence that mTOR deletion causes lethal, fully penetrant dilated cardiomyopathy (Zhang et al. 2010; Völkers et al. 2013). A study that inhibited the function of the mTOR kinase pharmacologically has shown that it has a negative effect on cardiomyocytes in vitro and in vivo after MI. Furthermore, using novel mTOR kinase inhibitors to treat cancer may result in cardiotoxicity in patients (Wander et al. 2011). These reports were consistent with our current findings, as the inhibited levels of mTOR in PD-intoxicated rats were improved when using protective therapy, particularly the combination treatment, which managed to raise mTOR close to normal levels.

In the current study, PD-intoxicated rats revealed several histopathological signs of cardiac muscles as wavy-shaped myofibers with unclear striation; some myofibers appeared necrotic, hemorrhage, and edema between cardiac myofibers. These findings agreed with Yang et al. (2021), who reported that rats intoxicated with PD at a dose of (2, 4, 6 mg/kg i.p.) revealed necrotic cardiac myofibers with hemorrhage in between. Moreover, our results were supported by Soudani and colleagues, who stated that PD-treated rats had damaged cardiac muscles with necrosis and hemorrhage and that the resulting lipid peroxidation from PD intoxication leads to membrane damage of cardiac myofibers causing heart injury (Soudani et al. 2011).

On the other hand, administration of BSS, TMZ, and a combination of both to PD-intoxicated rats resulted in a significant reduction of cardiac muscular degenerative changes as the cardiac myofibers appeared nearly normal elongated branched contained oval central nuclei, decreased the hemorrhage, edema, and necrosis till disappeared with the administration of both BSS and TMZ to PD-intoxicated rats. These results indicate the powerful antioxidant role of both BSS and TMZ against PD-induced cardiotoxicity. Our findings were supported by Sikandar et al., who stated that TMZ is a widely recommended antianginal agent with prominent antioxidant properties and cardioprotective benefits (Sikandar et al. 2020). Moreover, Koc et al. reported that BSS had protective effects against renal ischemia/reperfusion injury-induced myocardial cell damage (Koc et al. 2021).

## Conclusion

**Fig. 10:**
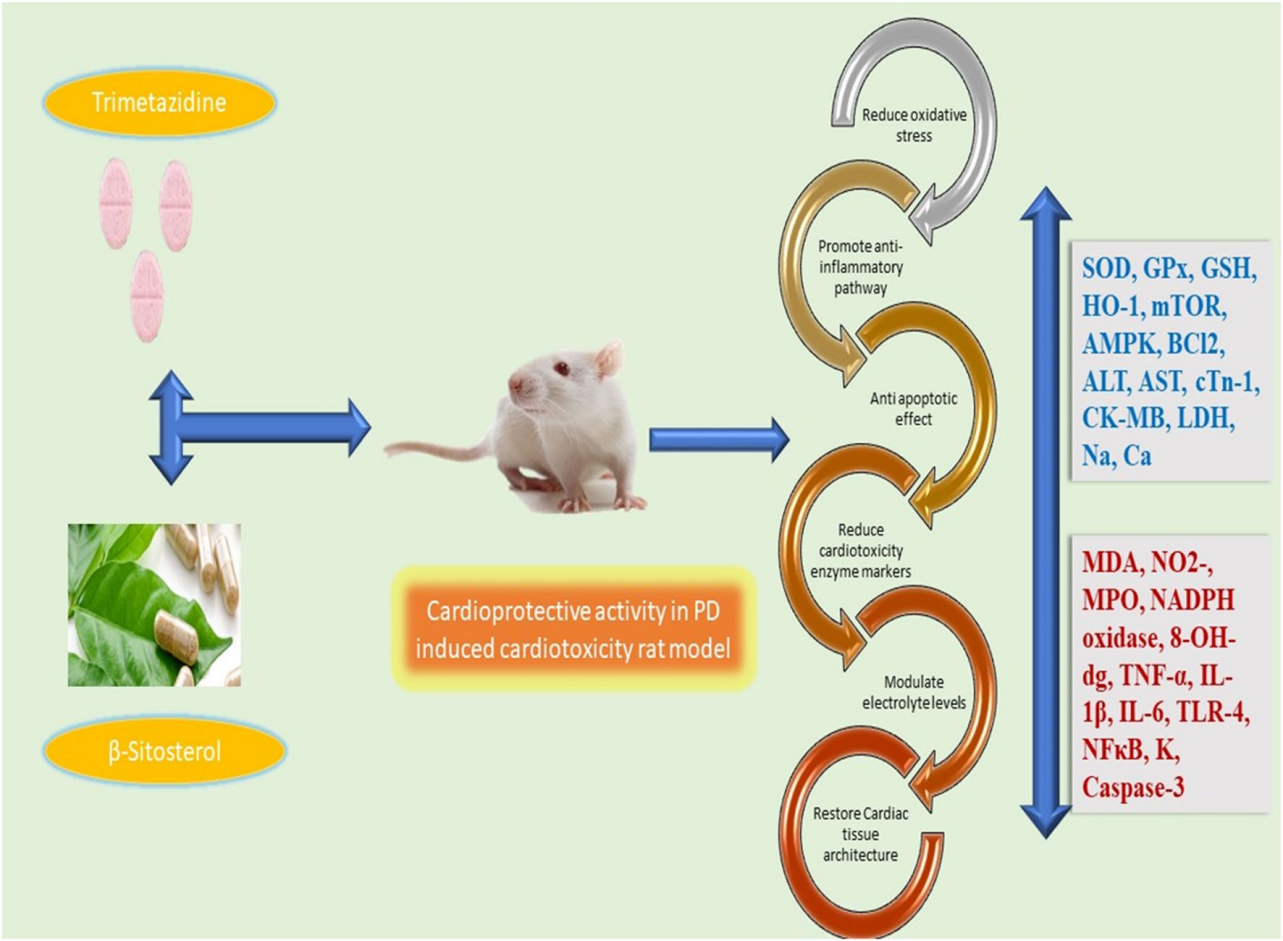
The schematic illustration of the possible signaling mechanisms by which β-sitosterol and trimetazidine mitigated the potassium dichromate-induced cardiotoxicity in rats

We have clarified the possible protective effects of BSS and TMZ combination therapy in rats against PD-induced cardiotoxicity. According to the present study, several ways to achieve this goal include reducing oxidative stress and inflammation. The study highlighted the ability of BSS + TMZ therapy to prevent apoptosis and restore the functionality of normal cardiac cells. It is interesting to note that the treatment has been associated with the modulation of the NF-κB/AMPK/mTOR/TLR4 and HO-1/NADPH signaling pathways, indicating improvement in cardiac function as well as the elimination of cardiotoxicity, as shown in Fig. 10.

## Data Availability

The datasets used and/or analyzed during the current study are available from the corresponding author on reasonable request. The data of this article are included within the article and its additional files.

## Abbreviations

PD: Potassium dichromate
BSS: β-Sitosterol
TMZ: Trimetazidine
TNF-α: Tumor necrosis factor-alpha
IL-6: Interleukin-6
IL-1β: Interleukin-1β
AMPK: AMP-activated protein kinase
mTOR: Mechanistic target of rapamycin
MDA: Malondialdehyde
GSH: Reduced glutathione
NO_2_^−^: Total nitrite end product
SOD: Superoxide dismutase
GPx: Glutathione peroxidase
NADPH: Nicotinamide adenine dinucleotide phosphate hydrogen
MPO: Myeloperoxidase
HO-1: Heme oxygenase 1
8-OHdG: 8-Hydroxydeoxyguanosine
TLR4: Toll-like receptor 4
ALT: Alanine aminotransferase
AST: Aspartate aminotransferase
CK-MB: Creatine kinase-myoglobin binding
LDH: Lactate dehydrogenase
BCL2: B-cell lymphoma2
cTn-1: Cardiac troponin-1

## References

[CR1] Anandasadagopan SK, Sundaramoorthy C, Pandurangan AK, Nagarajan V, Srinivasan K, Ganapasam S (2017). S-Allyl cysteine alleviates inflammation by modulating the expression of NF-κB during chromium (VI)-induced hepatotoxicity in rats. Hum Exp Toxicol.

[CR2] Atkinson C, He S, Morris K, Qiao F, Casey S, Goddard M, Tomlinson S (2010). Targeted complement inhibitors protect against posttransplant cardiac ischemia and reperfusion injury and reveal an important role in the alternative pathway of complement activation. J Immunol.

[CR3] Awoyomi OV, Adeoye YD, Oyagbemi AA, Ajibade TO, Asenuga ER (2021). Luteolin mitigates potassium dichromate-induced nephrotoxicity, cardiotoxicity, and genotoxicity through modulation of Kim-1/Nrf2 signaling pathways. Environ Toxicol.

[CR4] Banchroft GD, Steven A (1983). Theory and practice of histological technique.

[CR5] Bergmeyer HU, Hørder M, Rej R, (1986) International Federation of Clinical Chemistry (IFCC) Scientific Committee, Analytical Section: approved recommendation (1985) on IFCC methods for the measurement of catalytic concentration of enzymes. Part 3. IFCC method for alanine aminotransferase (L-alanine: 2-oxoglutarate aminotransferase, EC 2.6.1.2). J Clin Chem Clin Biochem. 24(7) 481–495 3734711

[CR6] Chandra R, Singh S, Ganguly C (2022) β-Sitosterol & quercetin enhances brain development in iodine deficient rat models. Nutr Health. 02601060221122209. 10.1177/02601060221122209 10.1177/02601060221122209 36017551

[CR7] Chen X, Li X, Zhang W, He J, Xu B (2018). Activation of AMPK inhibits inflammatory response during hypoxia and reoxygenation through modulating JNK-mediated NF-κB pathway. Metabolism.

[CR8] Chen X, Xia X, Dong T, Lin Z, Du L, Zhou H (2022). Trimetazidine reduces cardiac fibrosis in rats by inhibiting NOX2-mediated endothelial-to-mesenchymal transition. Drug Des Devel Ther.

[CR9] Chiorcea-Paquim AM (2022). 8-oxoguanine and 8-oxodeoxyguanosine biomarkers of oxidative DNA damage: a review on HPLC–ECD determination. Molecules.

[CR10] Datla SR, Dusting GJ, Mori TA, Taylor CJ, Croft KD, Jiang F (2007) Induction of heme oxygenase-1 in vivo suppresses NADPH oxidase–derived oxidative stress. Hypertension.50(4):636–642. 10.1161/HYPERTENSIONAHA.107.092296 10.1161/HYPERTENSIONAHA.107.092296 17679649

[CR11] Dézsi CA (2016). Trimetazidine in practice: review of the clinical and experimental evidence. Am J Ther.

[CR12] Ghosh P, Dey T, Bandyopadhyay D (2022) The pleiotropic role of melatonin against chromium-induced cardiovascular infirmities: a mechanistic insight. Melatonin Res. 5 (Sep. 2022), 209–253. 10.32794/mr112500130

[CR13] Görlach A, Bertram K, Hudecova S, Krizanova O (2015). Calcium and ROS: a mutual interplay. Redox Biol.

[CR14] Griendling KK, Touyz RM, Zweier JL, Dikalov S, Chilian W, Chen YR, Harrison DG, Bhatnagar A (2016). Measurement of reactive oxygen species, reactive nitrogen species, and redox-dependent signaling in the cardiovascular system. Circ Res.

[CR15] Gumede NM, Lembede BW, Brooksbank RL, Erlwanger KH, Chivandi E (2020). β-sitosterol shows potential to protect against the development of high-fructose diet-induced metabolic dysfunction in female rats. J Med Food.

[CR16] Hamdan DI, Tawfeek N, El-Shiekh RA, Khalil HMA, Mahmoud MY, Bakr AF, Zaafar D, Farrag N, Wink M, El-Shazly AM (2022). Salix subserrata bark extract-loaded chitosan nanoparticles attenuate neurotoxicity induced by sodium arsenate in rats in relation with HPLC–PDA-ESI–MS/MS profile. AAPS PharmSciTech.

[CR17] Hsu SM, Raine L, Fanger H (1981). The use of antiavidin antibody and avidin-biotin-peroxidase complex in immunoperoxidase technics. Am J Clin Pathol.

[CR18] Hu Y, Li J, Lou B, Wu R, Wang G, Lu C, Wang H, Pi J, Xu Y (2020). The Role of Reactive Oxygen Species in Arsenic Toxicity. Biomolecules.

[CR19] Huang BP, Lin CH, Chen HM, Lin JT, Cheng YF, Kao SH (2015). AMPK activation inhibits expression of pro-inflammatory mediators through downregulation of PI3K/p38 MAPK and NF-κB signaling in murine macrophages. DNA Cell Biol.

[CR20] Jiang F, Roberts SJ, Datla SR, Dusting GJ (2006). NO modulates NADPH oxidase function via heme oxygenase-1 in human endothelial cells. Hypertens.

[CR21] Kasirzadeh S, Ghahremani MH, Setayesh N, Jeivad F, Shadboorestan A et al (2021) *β*-sitosterol alters the inflammatory response in CLP rat model of sepsis by modulation of NF*κ*B signaling. BioMed Res. Int. 2021, e5535562. 10.1155/2021/5535562 10.1155/2021/5535562 PMC8105092 33997001

[CR22] Khalifa MMA, Bakr AG, Osman AT (2017). Protective effects of phloridzin against methotrexate-induced liver toxicity in rats. Biomed Pharmacother.

[CR23] Koc K, Geyikoglu F, Cakmak O, Koca A, Kutlu Z, Aysin F, Yilmaz A, Aşkın H (2021). The targets of β-sitosterol as a novel therapeutic against cardio-renal complications in acute renal ischemia/reperfusion damage. Naunyn Schmiedebergs Arch Pharmacol.

[CR24] Liu T, Zhang L, Joo D, Sun SC (2017). NF-κB signaling in inflammation. Signal Transduct Target Ther.

[CR25] Loizou S, Lekakis I, Chrousos GP, Moutsatsou P (2010). β-Sitosterol exhibits anti-inflammatory activity in human aortic endothelial cells. Mol Nutr Food Res.

[CR26] Manktelow A, Meyer AA (1986). Lack of correlation between decreased chemotaxis and susceptibility to infection in burned rats. J Trauma.

[CR27] Marklund SL (1985). Superoxide dismutase isoenzymes in tissues and plasma from New Zealand black mice, nude mice and normal BALB/c mice. Mutat Res.

[CR28] McMullen JR, Sherwood MC, Tarnavski O, Zhang L, Dorfman AL, Shioi T, Izumo S (2004). Inhibition of mTOR signaling with rapamycin regresses established cardiac hypertrophy induced by pressure overload. Circulation.

[CR29] Mehany HA, Abo-youssef AM, Ahmed LA, Arafa ESA, Abd El-Latif HA (2013). Protective effect of vitamin E and atorvastatin against potassium dichromate-induced nephrotoxicity in rats. Beni-Suef Univ J Basic Appl Sci.

[CR30] Mihara M, Uchiyama M (1978). Determination of malonaldehyde precursor in tissues by thiobarbituric acid test. Anal Biochem.

[CR31] Montgomery H, Dymock JJSP, Milton Rd, Cambridge, Determination of nitrite in water, ROYAL SOC CHEMISTRY THOMAS GRAHAM HOUSE. 1961

[CR32] Paglia DE, Valentine WN (1967). Studies on the quantitative and qualitative characterization of erythrocyte glutathione peroxidase. J Lab Clin Med.

[CR33] Rashad WA, Sakr S, Domouky AM (2022). Comparative study of oral versus parenteral crocin in mitigating acrolein-induced lung injury in albino rats. Sci Rep.

[CR34] Sahu BD, Koneru M, Bijargi SR, Kota A, Sistla R (2014). Chromium-induced nephrotoxicity and ameliorative effect of carvedilol in rats: involvement of oxidative stress, apoptosis and inflammation. Chem Biol Interact.

[CR35] Salama AAA, Mostafa RE, Elgohary R (2022). Effect of L-carnitine on potassium dichromate-induced nephrotoxicity in rats: modulation of PI3K/AKT signaling pathway. Res Pharm Sci.

[CR36] Schmitt DL, Curtis SD, Lyons AC, Zhang JF, Chen M, He CY, Mehta S, Shaw RJ, Zhang J (2022). Spatial regulation of AMPK signaling revealed by a sensitive kinase activity reporter. Nat Commun.

[CR37] Sedlak J, Lindsay RH (1968). Estimation of total, protein-bound, and nonprotein sulfhydryl groups in tissue with Ellman’s reagent. Anal Biochem.

[CR38] Senn AC, Kaegi R, Hug SJ, Hering JG, Mangold S, Voegelin A (2017). Effect of aging on the structure and phosphate retention of Fe(III)-precipitates formed by Fe(II) oxidation in water. Geochim Cosmochim Acta.

[CR39] Sikandar A, Farhat K, Afzal A, Ajmal K, Laeeq M, Khokhar A (2020). Protective effects of trimetazidine against doxorubicin-induced cardiotoxicity and hepatotoxicity in mice. J Ayub Med Coll Abbottabad.

[CR40] Soudani N, Troudi A, Bouaziz H, Ben Amara I, Boudawara T, Zeghal N (2011). Cardioprotective effects of selenium on chromium (VI)-induced toxicity in female rats. Ecotoxicol Environ Saf.

[CR41] Stohs SJ, Bagchi D (1995). Oxidative mechanisms in the toxicity of metal ions. Free Radic Biol Med.

[CR42] Taillé C, El-Benna J, Lanone S, Dang MC, Ogier-Denis E, Aubier M, Boczkowski J (2004). Induction of heme oxygenase-1 inhibits NAD(P)H oxidase activity by down-regulating cytochrome b558 expression via the reduction of heme availability. J Biol Chem.

[CR43] Tsioufis K, Andrikopoulos G, Manolis A (2015). Trimetazidine and cardioprotection: facts and perspectives. Angiology.

[CR44] Ussher JR, Keung W, Fillmore N, Koves TR, Mori J (2014). Treatment with the 3-ketoacyl-CoA thiolase inhibitor trimetazidine does not exacerbate whole-body insulin resistance in obese mice. J Pharmacol Exp Ther.

[CR45] Völkers M, Konstandin MH, Doroudgar S, Toko H, Quijada P, Din S, Joyo A, Ornelas L, Samse K, Thuerauf DJ, Gude N, Glembotski CC, Sussman MA (2013). Mechanistic target of rapamycin complex 2 protects the heart from ischemic damage. Circulation.

[CR46] Wander SA, Hennessy BT, Slingerland JM (2011). Next-generation mTOR inhibitors in clinical oncology: how pathway complexity informs therapeutic strategy. J Clin Invest.

[CR47] Wilcox CS (2005). Oxidative stress and nitric oxide deficiency in the kidney: a critical link to hypertension?. Am J Physiol Regul Integr Comp Physiol.

[CR48] Xiang HC, Lin LX, Hu XF, Zhu H, Li HP (2019). AMPK activation attenuates inflammatory pain through inhibiting NF-κB activation and IL-1β expression. J Neuroinflammation.

[CR49] Yang D, Yang Q, Fu N, Li S, Han B et al (2021) Hexavalent chromium induced heart dysfunction via Sesn2-mediated impairment of mitochondrial function and energy supply. Chemosphere 264(pt 2):128547. 10.1016/j.chemosphere.2020.128547 10.1016/j.chemosphere.2020.128547 33049514

[CR50] Yang Q, Han B, Xue J, Lv Y, Li S et al (2020) Hexavalent chromium induces mitochondrial dynamics disorder in rat liver by inhibiting AMPK/PGC-1α signaling pathway. Environ. Pollut. 265(pt A):114855. 10.1016/j.envpol.2020.114855 10.1016/j.envpol.2020.114855 32474337

[CR51] Ye Y, Bajaj M, Yang HC, Perez-Polo JR, Birnbaum Y (2017) SGLT-2 inhibition with dapagliflozin reduces the activation of the Nlrp3/ASC inflammasome and attenuates the development of diabetic cardiomyopathy in mice with type 2 diabetes. Further augmentation of the effects with saxagliptin, a DPP4 inhibitor. Cardiovasc Drugs Ther. 31(2):119–132. 10.1007/s10557-017-6725-2 10.1007/s10557-017-6725-2 28447181

[CR52] Zaafar D, Khalil HMA, Rasheed RA, Eltelbany RFA, Zaitone SA (2022) Hesperetin mitigates sorafenib-induced cardiotoxicity in mice through inhibition of the TLR4/NLRP3 signaling pathway. PLOS ONE. 17(8):e0271631. 10.1371/journal.pone.0271631 10.1371/journal.pone.0271631 PMC9362940 35944026

[CR53] Zhang D, Contu R, Latronico MVG, Zhang J, Rizzi R (2010). MTORC1 regulates cardiac function and myocyte survival through 4E-BP1 inhibition in mice. J Clin Invest.

[CR54] Zhang G, Ghosh S (2001). Toll-like receptor–mediated NF-κB activation: a phylogenetically conserved paradigm in innate immunity. J Clin Invest.

[CR55] Zhang H, Liu M, Zhang Y, Li X (2019). Trimetazidine attenuates exhaustive exercise-induced myocardial injury in rats via regulation of the Nrf2/NF-κB signaling pathway. Front Pharmacol.

[CR56] Zhang L, Ding W, Wang Z, Tang M, Wang F (2016). Early administration of trimetazidine attenuates diabetic cardiomyopathy in rats by alleviating fibrosis, reducing apoptosis and enhancing autophagy. J Transl Med.

[CR57] Zhen YZ, Lin YJ, Li KJ, Zhang GL, Zhao YF (2016). Effects of rhein lysinate on D-galactose-induced aging mice. Exp Ther Med.

[CR58] Zou H, Zhu XX, Ding YH, Jin QY, Qian LY (2017). Trimetazidine in conditions other than coronary disease, old drug, new tricks?. Int J Cardiol.

